# Efficacy of Periarticular Multimodal Drug Injection Without NSAIDs in Elderly Patients With Displaced Femoral Neck Fractures Undergoing Bipolar Hemiarthroplasty: A Prospective Triple-Blinded RCT

**DOI:** 10.7759/cureus.10271

**Published:** 2020-09-06

**Authors:** Jakrapong Orapin, Wuttichai Sutantavibul, Kulapat Chulsomlee, Chavarat Jarungvittayakon, Nachapan Pengrung, Norachart Sirisreetreerux, Noratep Kulachote, Tulyapreuk Tawonsawatruk, Pongsthorn Chanplakorn, Paphon Sa-Ngasoongsong

**Affiliations:** 1 Orthopedics, Faculty of Medicine Ramathibodi Hospital, Mahidol University, Bangkok, THA; 2 Orthopedics, Latyao Hospital, Nakhon Sawan, THA; 3 Chakri Naruebodindra Medical Institute, Faculty of Medicine Ramathibodi Hospital, Mahidol University, Samut Prakan, THA

**Keywords:** surgical site injection, perioperative pain management, postoperative analgesia, geriatric hip fracture, hip arthroplasty

## Abstract

Introduction

Recently, periarticular multimodal drug injection (PMDI) has demonstrated the ability to significantly reduce early postoperative pain with hip fractures in the elderly. Nonetheless, data on PMDI without non-steroidal anti-inflammatory drugs (NSAIDs) in these patients are still doubtful. The current study has evaluated the effect of PMDI with NSAIDs in elderly femoral neck fractures (FNFs) underlying bipolar hip arthroplasty (BHA).

Materials and methods

A prospective triple-blinded randomized controlled trial (RCT) was conducted in 28 elderly FNFs undergoing BHA. They were randomized into two groups: PMDI group (n=14), which received intraoperative PMDI (50-mL solution of 100-mg bupivacaine, 10-mg morphine, 300-mcg epinephrine, and 750-mg cefuroxime), and a placebo group (n=14), which received only saline solution. The primary outcome was a 10-point visual analog scale (VAS). Secondary outcomes were morphine consumption and cumulative ambulatory score (CAS), postoperative complications, and functional outcomes as a timed up-and-go (TUG) test and Harris hip score (HHS) at two, six, and 12 weeks postoperatively.

Results

The PMDI group demonstrated a significant reduction in the median VAS at the 48^th^ hour postoperatively as compared to the placebo group (P = 0.019), and a non-significant reduction in the median VAS at the 36^th^ and 60^th^ hours (P = 0.058 and 0.110, respectively) and in a median dosage of morphine consumption on the second postoperative day (P = 0.140). There was no significant difference in postoperative ambulation and functional outcome between both groups (P > 0.05, all).

Conclusion

The PMDI regimen without NSAIDs is effective for postoperative analgesia on the second postoperative day in elderly FNFs undergoing BHA without any significant difference in functional outcome or postoperative complications.

## Introduction

Elderly hip fracture is one of the major global health problems in the 21st century due to an increasing incidence with age and a high rate of morbidity and mortality [1]. Hip fractures are commonly accompanied by a substantial amount of pain that is significantly associated with many consequences such as delirium, depression, sleep disturbance, and decreased response to interventions for other disease states [2]. Moreover, poorly managed postoperative pain also prevents elderly patients from early mobilization, thereby decreasing their function as compared to the pre-injury ambulation status. As a result, those elderly patients with delayed ambulation have a high risk of cardiovascular and pulmonary complications, aggravations of comorbidities, worse functional outcomes, and postoperative mortality [3-4]. Therefore, effective perioperative pain management is one of the important goals in the treatment of hip fracture patients [5].

Regarding all hip fractures, femoral neck fractures account for approximately half, with the vast majority occurring in elderly patients after simple falls [6] and usually require surgical management to provide early ambulation and return patients to their pre-injury functional status [7]. In the elderly who sustain displaced femoral neck fracture, the recommended surgical option is hip arthroplasty due to significant improvement in subsequent reoperations, pain relief, and functional recovery as compared to internal fixation [8]. However, traditional pain management in patients undergoing hip fracture surgery is generally dependent on the use of opioids drugs, which not only provide suboptimal pain control but also result in the risk of common opioid-related side effects, such as oversedation and respiratory depression, which inhibit communication between patients and healthcare personnel and can delay ambulation and rehabilitation programs [9]. Recently, periarticular multimodal drugs injection (PMDI) for knee and hip arthroplasties have proven effective in reducing postoperative pain in hip arthroplasty by several systematic review and meta-analyses studies [10]. The most commonly used drugs for PMDI regimens include local anesthetics, opioids, NSAIDs, steroids, clonidine, epinephrine, and antibiotics. However, PMDI regimens in hip arthroplasty are varied in previous literature, and there is no consensus on the dosage and composition of the injected drugs. Moreover, NSAIDs are not recommended for perioperative pain management in elderly hip fracture patients due to the high risk of NSAIDs-related complications such as upper gastrointestinal bleeding, nephrotoxicity, and fluid retention [11]. Furthermore, previous studies have shown that the use of 30-mg ketorolac in the PMDI regimen for hip arthroplasty resulted in a mean maximal plasma concentration comparable to an intramuscular injection of 10-mg ketorolac and may not be safer than the other routes of administration [12-13], thereby requiring a similar restriction for patients at risk for developing side effects. Therefore, the routine PMDI regimen with NSAIDs might not be applicable in clinical practice, especially in elderly patients at risk for NSAIDs-related complications. The aim of this study was to evaluate the effectiveness of PMDI without NSAIDs in elderly patients with displaced FNFs undergoing BHA for postoperative pain reduction, such as the 10-point visual analog scale (VAS) in the early postoperative period, as compared to those who receive a placebo injection.

## Materials and methods

Study design and inclusion and exclusion criteria

This study was designed as a prospective triple-blinded randomized placebo-controlled trial in a medical university hospital and the study protocol was approved by the Ethical Clearance Committee on Human Rights to Research Involving Human Subjects (Protocol ID 07-59-10). The manuscript was prepared according to the Consolidated Standards of Reporting Trials (CONSORT) guideline [14]. The study participants were those who were diagnosed as a closed fracture of the femoral neck and were scheduled for bipolar hemiarthroplasty in Ramathibodi Hospital during October 2017 and December 2019. The inclusion criteria were patients who (1) had a displaced femoral neck fracture, (2) were age 60 years or older, (3) were scheduled for bipolar hemiarthroplasty, (4) had preinjury independent ambulatory status without gait aids, and (5) were willing to follow the study’s protocol and given their consent. The exclusion criteria were the patients who (1) had multiple trauma or fractures, (2) had a previous hip fracture or surgery, (3) had an underlying neuromuscular disorder or cognitive impairment that affected muscle function or made them unable to follow the postoperative protocol, (4) had a pathologic fracture other than osteoporosis such as a metastatic fracture, (5) had a contraindication or allergy to the drug in the PMDI protocol, (6) were unable to follow the postoperative protocol, and (7) who wanted to withdraw by themselves.

Sample size calculation and randomization method

The sample size was estimated by using the web-based version of the PS: Power and Sample size calculation program 3.1.6 (https://statcomp2.app.vumc.org/ps/; Vanderbilt University, TN) and the data review from our pilot study in 20 elderly FNF patients who underwent BHA, as the mean and standard deviation of 36th hour postoperative VAS was 2.4 ± 1.1. Assuming that PMDI without NSAIDs should reduce VAS more than 50% as compared with the control group and setting the pre-study power of the test (β) as 0.8, the significant difference (α) as 0.05, and the allocation ratio as 1:1. Therefore, the sample size of each group was 14 patients. The blocked-randomization was generated by STATA 12.0 software (Stata Corp, College Station, TX), with a block size of 4 and further concealed with sealed envelopes in a sequentially numbered container. The envelopes were sequentially opened intraoperatively before prosthesis implantation by a research assistant nurse who was not involved in outcome assessment. Then, all patients were allocated to one of two groups, the PMDI group or the placebo group.

Drug preparation and administration method

**Figure 1:**
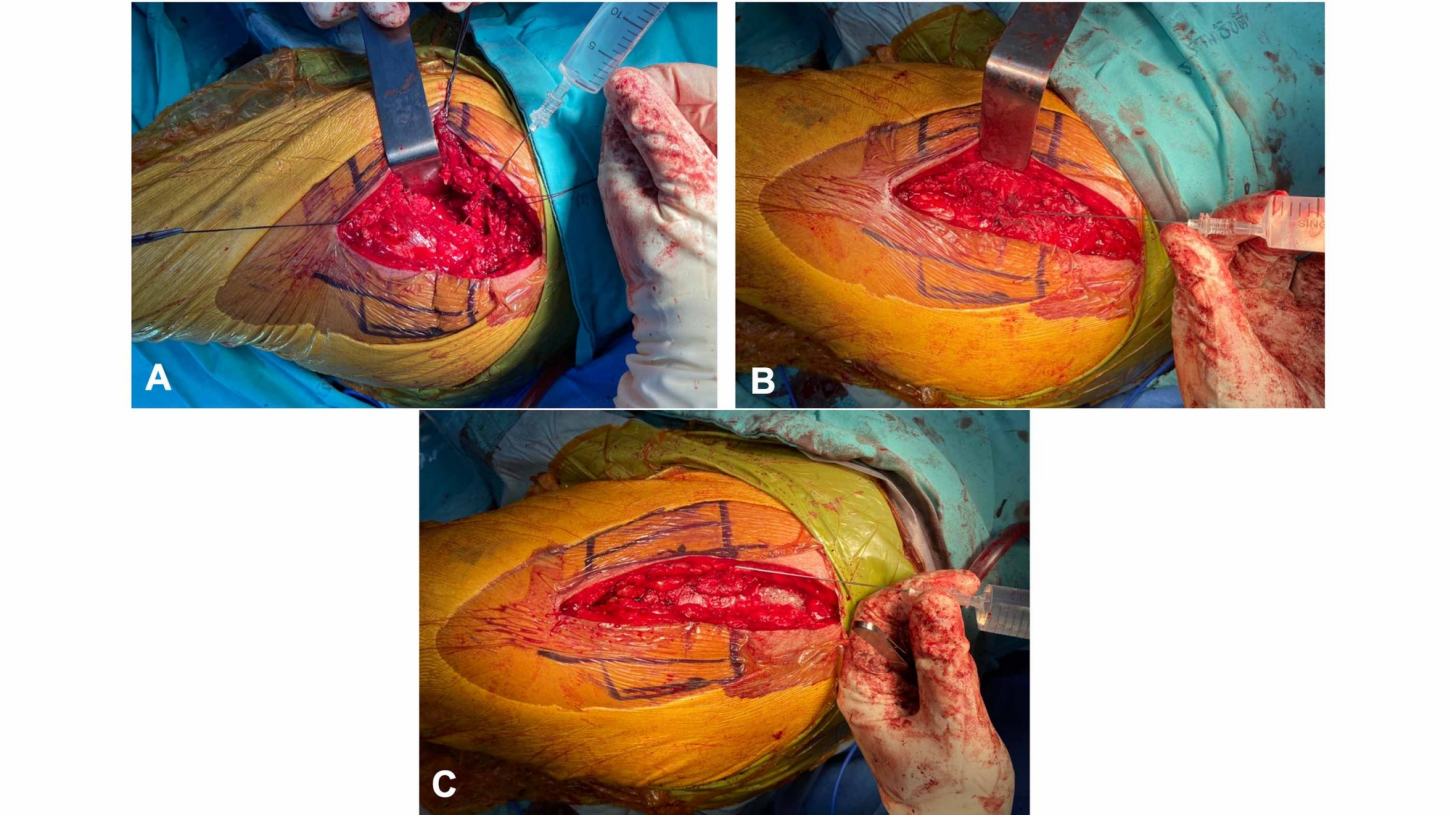
Demonstration of intraoperative peri-articular multimodal drug injection Before the final implant insertion, the prepared solution was injected at the hip capsule, starting from the lateral capsule to the posterior, medial, and anterior capsules in a clockwise and anti-clockwise pattern (A). Then, after the prosthesis insertion and gluteus medius repair, the solution was injected at the muscle and tensor fascia lata (B). Finally, the solution was injected in the subcutaneous tissue before skin closure (C).

In both the PMDI and placebo groups, the injected solution was prepared by a research assistant as a total of 50 mL volume, the same-size syringe under sterile conditions, behind an opaque screen that blinds the surgeon and the outcome assessor. In the PMDI group, the solution contained 100 mg bupivacaine, 10 mg morphine sulfate, 300 mcg adrenaline, 750 mg cefuroxime, and physiologic saline. In the placebo group, the solution was only physiologic saline. Both prepared solutions had the same appearance. The solution was injected with the same pattern, starting at the joint capsule (as rotating in a clockwise or counter-clockwise pattern from the lateral joint capsule to the posterior, the medial, and the anterior joint capsules, respectively), the gluteus muscle, and the subcutaneous layer (Figure 1).

Surgical procedure

All operations were performed by a single orthopedic surgeon (PS) who had experience in hip fracture surgery of 10 years or longer, using the same surgical approach as the anterolateral hip approach with anterior hemimyotomy [15] and the standard surgical technique following the prosthesis instruction. After spinal anesthesia without a specific nerve block and indwelling urinary catheter insertion were performed, the patients were then placed in the lateral decubitus position under sterile conditions. The incision was 10-12 cm in length on the anterior border of the femur, starting from 6 cm proximal to the tip of the greater trochanter and then distal along the femoral axis. The tensor fascia lata was incised longitudinally, and then the anterior half of the gluteus medius was sutured (for resuture at the end of the operation) and cut at 1 cm proximal to its insertion. The anterior hip capsule was identified, incised as an inverted-T shape, and sutured (for closure at the end of the operation). The femoral neck was identified and osteotomized by using the prosthesis template. The femoral head was then removed and measured to determine the size of the bipolar head prosthesis. Then, the leg was placed in 90-degree flexion and 90-degree external rotation. The superolateral femoral neck was removed followed by femoral stem preparation, starting from the smallest size to the largest size, as possible. Then, the femoral head trial was inserted, and the hip was reduced. The decision on hip prosthesis, cemented or cementless femoral stem, was based on proximal femur morphology. Proper prosthesis size (both femoral head and stem size) and prosthesis neck length were determined by intraoperative fluoroscopy and implant stability. Cementless femoral stem (Corail® Hip Stem, DePuy Synthes, Johnson & Johnson Medical Devices, NJ) was chosen in patients with good bone quality (such as Dorr type A or B) and good stability with a trial component. In cases of osteoporotic bone (such as Dorr type C) or inability to achieve stable fixation with a trial component, a cemented femoral stem (C-stem® AMT, DePuy Synthes) is used with antibiotic-loaded bone cement (Palacos® Bone Cement, Zimmer Inc., Warsaw, IN). A standard drain tube (size 8 Redon drain, B-Braun Ltd., Melsungen, Germany) is placed anterior to the hip capsule, exited distally to the surgical wound, and connected to a high-pressure vacuum drain (Drainobag® 600V Lock, B-Braun, Melsungen AG, Germany). The gluteus medius muscle and tensor fascia lata were repaired with 1-0 Vicryl (Ethicon, Cincinnati, OH). Subcutaneous tissue and skin were then closed layer by layer using 2-0 Vicryl (Ethicon) and 3-0 Ethilon® (Ethicon). All wound closures were performed using the interrupted mattress suture technique. The drain clamp was opened immediately after wound closure.

Postoperative care 

Postoperative care and rehabilitation were managed by the same protocol. After surgery, all patients were admitted into the orthopedic ward and routinely managed by the orthogeriatric nurse team. The hemoglobin level was measured immediately in the ward and on the first and second postoperative days. The blood transfusion protocol for the patients with and without a cardiac arrest was considered when hemoglobin (Hb) was less than 10 gm% and 8 gm%, respectively, or the patient had positive anemic symptoms (dyspnea, tachypnea, hypoxemia) [16]. Postoperative fluid administration was managed by the trauma experts’ team to ensure adequate fluid balance. The intravenous fluid was discontinued as soon as possible after the patients were able to have adequate oral fluids and urine output. Intravenous and urinary catheters were generally removed on the second postoperative day to allow early mobilization. All patients were encouraged to mobilize into a sitting position at the bedside within 12 hours postoperatively and to perform ankle, knee, and hip muscle exercises as soon as possible to prevent a venous thromboembolism complication. Then, they were proceeded to stand and walk with gait aid and weight-bearing as tolerated within three days postoperatively.

Pain management protocol

All patients received the same postoperative pain management protocol, including both intravenous and oral pain medications. During the three-day postoperative period, pain intensity was assessed immediately after arriving at the orthopedic ward and then every two hours (or when the patient complained) by using 10-point VAS. Intravenous 3 mg morphine sulfate every 4 hours PRN for pain score >3 was prescribed. If the pain score did not decrease to less than 3 after 60 minutes, rescue analgesia with intravenous 1.5 mg morphine was added. The oral pain medications consist of acetaminophen 500 mg every six hours for three days and Duocetz® (paracetamol 325 mg and tramadol 37.5 mg) (Mega Lifesciences, Thailand) one tablet every eight hours. If the patient experienced the side effect of opioids medication, such as nausea and vomiting, intravenous 4-mg ondansetron was then administered.

Data collection and outcome measurement

The following demographic data were collected preoperatively by a research assistant: age, gender, body mass index, underlying disease, side of operation, American Society of Anesthesiologists (ASA) physical status, side of injury, time from admission to surgery, and preoperative laboratory values (hemoglobin, platelet count, glomerular infiltration rate, prothrombin time, partial thromboplastin time, international normalized ratio, and serum albumin level). Perioperative data were recorded: type of femoral stem (cemented or cementless), operative time, estimated blood loss, blood transfusion, and length of hospital stay. Postoperative complications related to hip fracture and hip arthroplasty were collected, including death, surgical complication (intraoperative fracture, vascular injury, and wound complication), and medical complication (delirium, infection, cardiac complication, pulmonary complication, venous thromboembolic events, gastrointestinal complication, urinary tract complication, and pressure ulcers) [17-18]. Postoperative pain outcomes were assessed by two methods: (1) 10-point visual analog scale (VAS) measurement is assessed by using a 10-cm line marked at every 1 cm increment with the description ‘no pain’ on the left end and ‘worst pain’ on the right end. Subsequent recordings of VAS were performed on separate sheets of paper to prevent patients from comparing scores with previous ones. VAS was measured in the preoperative period and again at 12, 24, 36, 48, and 60 hours postoperatively. (2) Morphine consumption is assessed by summation of the total amount of morphine use (in mg) on the first, second, and third postoperative days. Postoperative in-hospital ambulation or basic mobility was assessed by using the cumulative ambulation score (CAS) that rates the patient’s independence with regard to three activities (getting in and out of bed, sit-to-stand-to-sit from a chair, and walking). Each activity is assessed on a three-point ordinal scale from 0 to 2, resulting in a total daily CAS score ranging from 0 to 6 (Table 1) [19].

**Table 1:** Cumulative ambulatory score (CAS)

Activity	Not able to do, despite human assistance or verbal cueing	Able to do with human assistance or verbal cueing	Able to do safely without human assistance or verbal cueing
Get in and out of bed	0	1	2
Sit-to-stand-to-sit from chair	0	1	2
Walking	0	1	2

Postoperative functional outcomes were assessed by two methods, as the Timed Up-and-Go (TUG) test and Harris Hip Score (HHS) at two, six, and 12 weeks postoperatively. The TUG test is defined as the time in seconds measured as the patient rises from an armchair, walks for 3 meters, turns, walks back, and sits down again [20]. HHS is a clinician-based outcome measure administered by a qualified health care professional (such as a physician or physical therapist) for the assessment of the outcome after hip surgery in the adult population. HHS gives a maximum of 100 points and consists of four clinical domains (best possible outcome) covering pain (1 item, 0-44 points), function (7 items, 0-47 points), absence of deformity (1 item, 4 points), and range of motion (2 items, 5 points) [21].

Statistical analysis

Statistical analysis was performed using MedCalc Statistical Software version 15.8 (MedCalc Software bvba, Ostend, Belgium). A Kolmogorov-Smirnov test was used to determine the normality of the data. Normally distributed continuous data were calculated as mean ± standard deviation (SD) and compared using the student’s t-test. Non-normally distributed continuous data were calculated as the median (range) and compared using the Mann-Whitney U test. Categorical variables were compared with chi-square or Fisher’s exact test as appropriate. A P-value <0.05 was considered statistically significant. The intention-to-treat analysis was applied.

## Results

**Figure 2:**
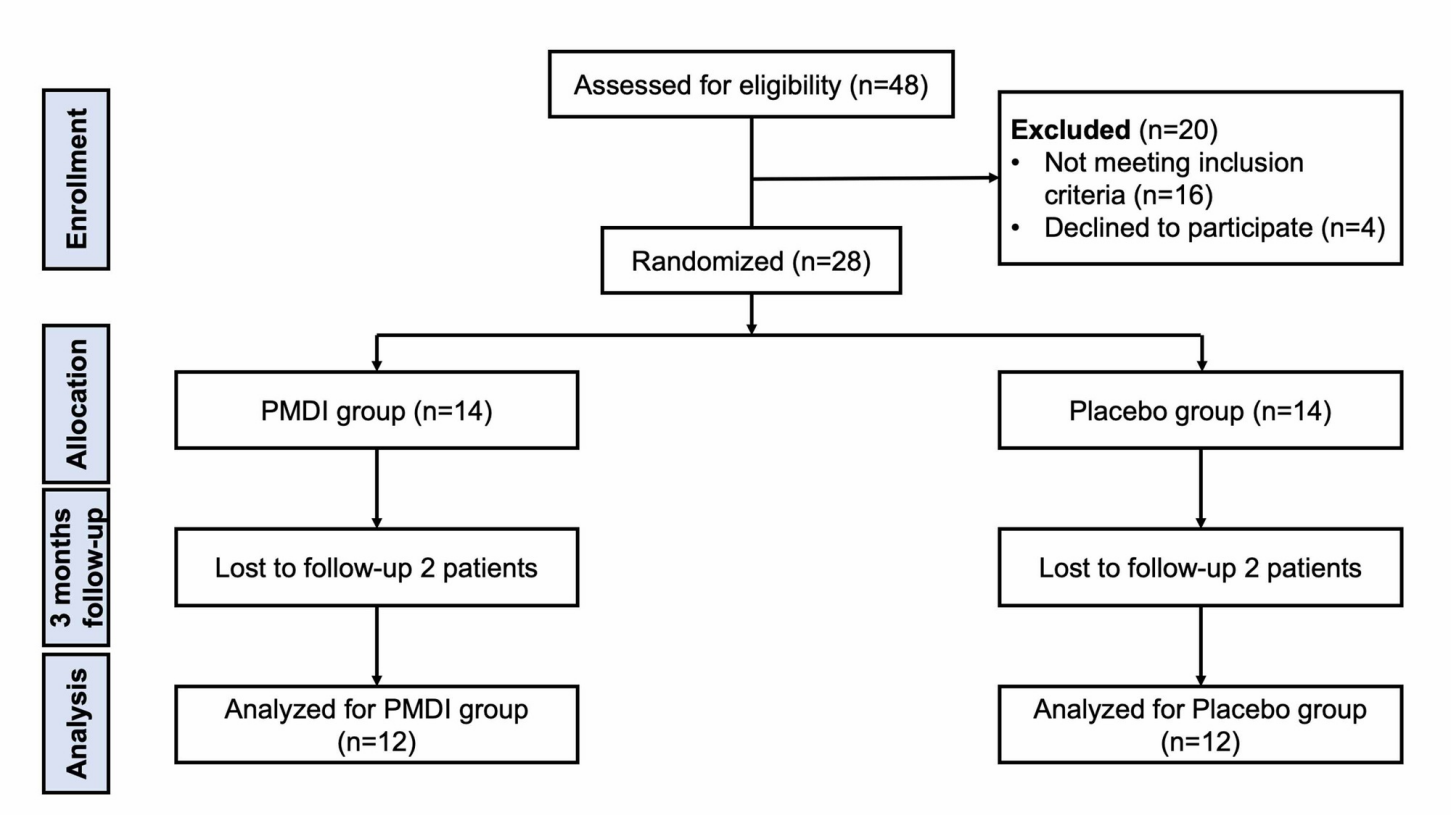
Flow diagram of this study

A total of 28 patients were randomly assigned to the PMDI and placebo groups (n=14 each). All patients followed the same postoperative management and rehabilitation protocol. Two patients in each group were lost to follow-up after discharge (one patient in each group was lost to follow-up at two weeks and six weeks, respectively). Therefore, there were 12 patients in each group for the final evaluation (Figure 2).

The demographic and preoperative laboratory data from all patients are presented in Table 2. The average age of the PMDI group and the placebo group was 77 years (range 65-86 years) and 73 years (range 62-88 years), respectively. Female gender accounted for 93% in the PMDI group (n = 13) and for 79% in the placebo group (n = 11). The majority of the ASA physical status in these elderly patients was grade 3, as 71% in the PMDI group (n = 10) and 50% in the placebo group (n = 7), respectively. The median time from admission to surgery was one day in both groups (range 0-12 days in the PMDI group, 0-3 days in the placebo group). One patient (7%) in the PMDI group had the preoperative complication of high-grade fever and was diagnosed with an influenza infection, which required anti-viral medication and delayed surgery for 10 days. However, both groups showed no significant difference in demographic data such as age, gender, body mass index, ASA grading, side of injury, time between admission date and surgical date, and preoperative laboratory values (hemoglobin, platelet count, glomerular filtration rate, prothrombin time, partial thromboplastin time, international normalized ratio, and albumin) (P > 0.05 for all).

**Table 2:** Demographic and preoperative laboratory data ℇ value presented as mean ± standard deviation, ∆ value presented as number of cases (percentage), ∞ value presented as a ratio of cases having that condition a calculated with the student's t-test, b calculated with Fisher’s exact test, BMI = body mass index, ASA = American Society of Anesthesiologists, Hb = hemoglobin, GFR = glomerular filtration rate, PT = prothrombin time, PTT = partial thromboplastin time, INR = international normalized ratio

	PMDI group (n = 14)	Placebo group (n = 14)	P-value
Age, year^ℇ^	77 ± 7	73 ± 8	0.205^a^
Female gender^∆^	13 (93%)	11 (79%)	0.596^b^
BMI, kg/m^2ℇ^	22.4 ± 3.8	22.4 ± 3.2	0.950^a^
ASA grade 2 : 3 : 4^∞^	3 : 10 : 1	5 : 7 : 2	0.506^b^
Right side injury^∆^	9 (64%)	5 (36%)	0.257^b^
Time to surgery, day^ℇ^	1 (0-12)	1 (0-3)	0.400^b^
Preoperative laboratory values			
Hb, g/dL^ℇ^	12.0 ± 1.6	12.2 ± 1.6	0.679^a^
Platelet count, x10^3^/mm^3^	233 ± 77	250 ± 91	0.592^a^
GFR, mL/min^ℇ^	77 ± 20	72 ± 27	0.551^a^
PT, second^ℇ^	12.0 ± 0.9	12.0 ± 0.5	0.921^a^
PTT, second^ℇ^	26.1 ± 3.7	25.1 ± 2.0	0.356^a^
INR^ℇ^	1.01 ± 0.09	1.00 ± 0.05	0.732^a^
Albumin, g/dL^ℇ`^	33.7 ± 5.7	33.4 ± 4.5	0.872^a^

The perioperative data are presented in Table 3. Cementless was used in 57% of the PMDI group and 64% of the placebo group. The average operative time was 96 minutes (range, 45-140 minutes) in the PMDI group and 98 minutes (range, 50-135 minutes) in the other. The average estimated blood loss was 243 mL (range, 50-700 mL) in the PMDI group and 250 mL (range, 50-500 mL) in the placebo group. Two patients in each group received packed red cell transfusion (one patient in each group received one unit, while one patient in each group received two units). The PMDI group showed a non-significant difference in perioperative data, as the type of femoral stem, operative time, estimated blood loss, blood transfusion, and length of hospital stay, as compared to the placebo group (P > 0.05 for all).

**Table 3:** Perioperative data ∞ value presented as a ratio of cases having that condition, ℇ value presented as mean ± standard deviation a calculated with the student's t-test, b calculated with the Fisher’s exact test, PRC = packed red cell, PMDI = periarticular multimodal drug injection

	PMDI group (n = 14)	Placebo group (n = 14)	P-value
Cemented : Cementless^∞^	6:8	5:9	1.000^b^
Operative time, min^ℇ^	96 ± 23	98 ± 24	0.827^a^
Estimated blood loss, m ^ℇ^	243 ± 116	250 ± 178	0.901^a^
PRC transfusion, unit^ℇ^	0 (0-2)	0 (0-2)	1.000^b^
Length of hospital stay, day^ℇ^	4.5 (3-15)	6.5 (3-14)	0.674^b^

Pain outcome

**Figure 3:**
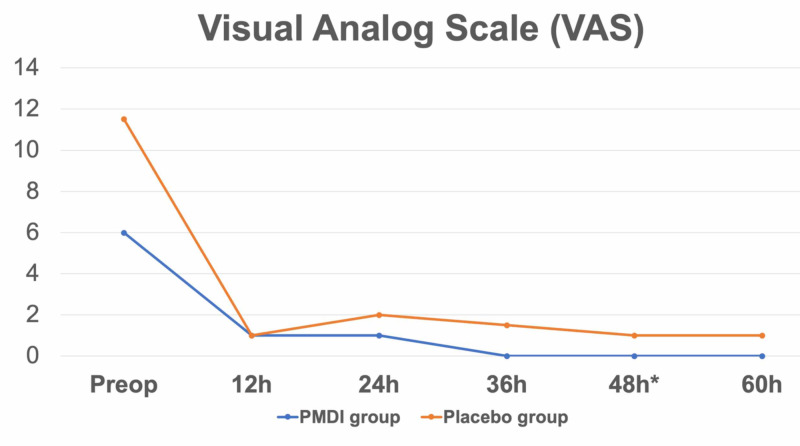
Visual analog scale (VAS) outcome The PMDI group shows a significant improvement in pain reduction as compared to the placebo group at 48 hours postoperatively (*), PMDI = periarticular multimodal drug injection

Regarding the VAS outcome, the comparison within the same group in both the PMDI and placebo groups showed a significant improvement in 10-point VAS, as the median preoperative VAS significantly reduced from 6.0 and 5.5 to 0.0 and 1.0 in the PMDI and placebo groups, respectively (P < 0.001 both). For the comparison between both groups, there was no significant difference in the median VAS in the preoperative period and 12 hours and 24 hours postoperatively (P < 0.05 all). However, the PMDI group demonstrated a significant reduction in the median VAS at the 48th hour (0.0, range 0-3) as compared to the placebo group (1.0, range 0-7; P = 0.019). Moreover, the PMDI group also showed a non-significant trend of pain reduction compared to the placebo group at 36 and 60 hours postoperatively. At the 36th hour postoperatively, the median VAS in the PMDI group was 0.0 (range 0-3) and those in the placebo group was 1.5 (range 0-9, P < 0.058). Likewise, the median VAS at the 60th hour in the PMDI group was 0.0 (range 0-5) and those in the placebo group was 1.0 (range 0-5, P < 0.110) (Figure 3).

Concerning the morphine consumption, the PMDI group showed a significant reduction in the median dosage of morphine consumption, from 2.5 mg (range 0.0-12.0 mg) on the first postoperative day to 0.0 mg (range 0.0-3.0 mg) on the third postoperative day (P < 0.001). However, the placebo group revealed only a non-significant trend in the reduction of morphine consumption, as from 1.5 mg (range 0.0-27.0 mg) in the first postoperative day to 0.0 (range 0.0-6.0 mg) on the third postoperative day (P = 0.057). By comparing both groups, there was no significant difference in the median dosage of morphine consumption in the first and the third postoperative day (P = 0.853 and 0.571, respectively). However, the PMDI group was non-significantly lower in the median dosage of morphine consumption than the placebo group on the second postoperative day (0.0 mg versus 1.0 mg, P < 0.140) (Table 4).

**Table 4:** Postoperative morphine consumption ∅ value presented as median (range): 1. P-value calculated from repeated measure ANOVA, 2. P-value calculated from the Wilcoxon rank-sum test; PMDI = periarticular multimodal drug injection, ANOVA = analysis of variance

Morphine consumption	PMDI group^∅^ (n = 14)	Placebo group^∅^ (n = 14)	P-value^2^
Day 1	2.5 (0.0-12.0)	1.5 (0.0-27.0)	0.853
Day 2	0.0 (0.0-6.0)	1.0 (0.0-9.0)	0.140
Day 3	0.0 (0.0-3.0)	0.0 (0.0-6.0)	0.571
P-value^1^	<0.001*	0.057	

Postoperative complication

The postoperative complications are presented in Table 5. One patient in the PMDI group had postoperative sciatic nerve palsy and was treated with a conservative method (transcutaneous electrical nerve stimulation (TENS) and a brace). Two patients in the placebo group had postoperative complications of fever due to urinary tract infection and postoperative new-onset atrial fibrillation with delirium. They were successfully treated with intravenous antibiotics and correction of electrolyte imbalance. The length of hospital stay in these patients was four days (one patient in the PMDI group), and 13 and seven days (two patients in the placebo group), respectively. Therefore, the overall complication rate was 8.3% (n = 1) in the PMDI group and 16.7% (n = 2) in the placebo group.

**Table 5:** Postoperative complication ∆ value presented as number of cases (percentage), PMDI = periarticular multimodal drug injection

Complication	PMDI group (n = 12)	Placebo group (n = 12)	P-value
Overall complication^∆^	1	2	1.000
Complication^∆^			
New-onset cardiac arrhythmia	0	1	0.262
Infection	0	1	
Delirium	0	1	
Nerve injury	1	0	

Postoperative ambulation and functional outcome

The average CAS on the first and second postoperative days and the median CAS on the third postoperative days in the PMDI group were 0.9±0.8, 3.4±1.1, and 6.0 (range 3-6), whereas those in the placebo group were 0.6±0.6, 3.3±1.1, and 4.5 (range 2-6). Both the PMDI and placebo groups showed significant improvement in the mean CAS within the same group at all assessment time points (P < 0.001 both). However, there was no significant difference in the CAS between both the PMDI and placebo groups at all assessment time points during this three-day postoperative period (P > 0.05 all). There was also no significant difference in the proportion of the elderly patients who had independent basic mobility (CAS = 6) at discharge (8 patients in the PMDI group versus 5 patients in the placebo group, P = 0.449) (Table 6).

**Table 6:** Postoperative cumulative ambulatory score (CAS) ℇ value presented as mean ± standard deviation, ∅; value presented as median (range), 1. P-value calculated from repeated measure ANOVA, 2. P-value calculated from the Wilcoxon rank-sum test, PMDI = periarticular multimodal drug injection

CAS	PMDI group (n = 12)	Placebo group (n = 12)	P-value^2^
Day 1^ℇ^	0.9 ± 0.8	0.6 ± 0.6	0.298
Day 2^ℇ^	3.4 ± 1.1	3.3 ± 1.1	0.729
Day 3^∅^	6.0 (3-6)	4.5 (2-6)	0.312
P-value^1^	<0.001*	<0.001*	

Both the PMDI and placebo groups demonstrated significant improvement in the postoperative functional outcomes, as measured by TUG score and HHS, within the same group at all assessment time points (P < 0.01 all). However, there was no significant difference in the TUG score and HHS between both PMID and placebo groups at all assessment time points during this 12-week postoperative period (P > 0.05 all) (Table 7).

**Table 7:** Postoperative functional outcome ℇ value presented as mean ± standard deviation, 1. P-value calculated from repeated measure ANOVA, 2. P-value calculated from the student's t-test, TUG = Timed Up-and-Go, HHS = Harris Hip Score, PMDI = periarticular multimodal drug injection

Functional outcome	PMDI group^ℇ ^ (n =12)	Placebo group^ℇ ^ (n =12)	P value^2^
TUG score			
2 weeks	84.7 ± 26.7	88.1 ± 34.3	0.785
6 weeks	51.0 ± 17.2	48.5 ± 16.5	0.720
12 weeks	24.8 ± 5.3	31.7 ± 15.4	0.167
P-value^1^	<0.001*	0.001*	
HHS			
2 weeks	58.5 ± 11.1	53.1 ± 12.8	0.282
6 weeks	73.9 ± 9.1	72.6 ± 5.9	0.676
12 weeks	81.9 ± 7.5	81.7 ± 5.4	0.927
P-value^1^	<0.001*	<0.001*	

## Discussion

Adequate perioperative pain control is one of the most important goals in the management of elderly hip fracture surgery. The multimodal analgesia approach offers better postoperative pain control as compared to traditional pain management with opioids alone and makes postoperative recovery faster [22]. Recently, PMDI has been proven effective in postoperative pain reduction in hip arthroplasty [10]. However, PMDI regimens in the previous studies were still varied in dosage and drug composition [10], and there were only a few studies demonstrating the effectiveness of PMDI in hip fracture surgery [23-24]. In addition, the use of NSAIDs in the elderly hip fracture is generally prohibited due to the risk of life-threatening complications. The present study aimed to evaluate the effectiveness of PMDI without NSAIDs for postoperative pain control in elderly patients with displaced femoral neck fracture who underwent bipolar hip arthroplasty as compared with a placebo injection. The results of this research are reported through the assessment of 10-point VAS, morphine consumption, and CAS during the 72-hour postoperative period and the evaluation of the Timed Up-and-Go test and the Harris Hip Score for 12 weeks after surgery.

A comparison of the median score of 10-point VAS (assessed preoperatively and postoperatively every 12 hours from 12 hours to 60 hours) and the median amount of morphine use (assessed postoperatively at 24 hours, 48 hours, and 72 hours) indicated significant postoperative pain reduction at the 48th hour and the non-significant postoperative pain reduction at the 36th and 60th hours and the non-significant reduction in morphine use during the postoperative period between the 24th and 48th hours in the PMDI group as compared to the placebo group (Figure 3 and Table 4). These findings reveal that the application of PMDI in elderly femoral neck fracture patients who received bipolar hip arthroplasty is effective for postoperative pain control without any significant difference in postoperative complications (Table 5). This could provide the supplementary postoperative analgesia effect until the third postoperative day, which is comparable to the results from the previous meta-analysis study by Jimenez-Almonte et al., on the local infiltration analgesia in elective total hip arthroplasty [25]. Regarding the results from this study and the previous studies on PMDI in hip fracture. Our findings on the additional benefits of PMDI are comparable to the results of previous studies by Kang et al. [23] and Koehler et al. [24]. In 2013, Kang et al. presented a single-blinded randomized controlled trial (RCT) that studied the effect of multimodal pain management in 82 elderly hip fracture patients undergoing bipolar hemiarthroplasty by comparing between those who received combined preemptive medication (10 mg oxycodone SR and 200-mg celecoxib) with intraoperative periarticular injection (100 mL cocktail solution of 300 mg ropivacaine, 10 mg morphine sulfate, 30 mg ketorolac, 300 -mcg epinephrine, 1000 mg cefmetazole, and physiologic saline) (n = 43) and those who did not receive (n = 39). They showed that the intervention group had significantly lower pain on postoperative days one and four, and a significantly lower amount of fentanyl and frequency of use of patient-controlled analgesia as compared to the control group [23]. Similarly, in 2017, Koehler et al. (p.512) also presented a double-blinded RCT that studied the effect of multimodal drug injection in the 102 patients who had a femoral fracture and underwent surgical fixation or hip arthroplasty by comparing between those who received PMDI (100 mL cocktail solution of 400 mg ropivacaine, 600 mcg epinephrine, 5 mg morphine sulfate, and physiologic saline) (n = 47) and those who did not receive (n = 55). They demonstrated that the intervention group had significantly lower VAS scores during the first 12-hour postoperative period and significantly lower narcotic consumption in the first eight-hour postoperative period as compared to the control group. Although the findings of the significant reduction on VAS and narcotic consumption between this study and the previous studies are not in the same postoperative period, this could be explained by the difference of research methodology and the effect of NSAIDs. Due to the strict perioperative protocol, the present study was designed the anesthesia protocol as spinal anesthesia while the previous studies used both general anesthesia and regional anesthesia [23] or only general anesthesia [24]. Therefore, in this study, the effect of spinal anesthesia could preclude the effect of PMDI in the early postoperative period as shown as the non-significant difference in the VAS and narcotic consumption during the first 24 hours after surgery. Moreover, the addition of NSAIDs in the PMDI regimen in the previous study (Kang et al., 2013, p.291) should theoretically reduce the postoperative pain and inflammation from the surgical trauma resulting in longer postoperative pain control as prolonged as four days after surgery.

Both the PMDI and placebo groups showed a significant improvement after surgery (within the same group) and a non-significant difference in the postoperative recovery of basic mobility (between both groups), which was defined by CAS on the first, second, and third postoperative days (Table 6). However, the PMDI group demonstrated a non-significant higher median CAS score on the third postoperative day (6.0, range 3-6) and a non-significant higher proportion of elderly patients who had returned to their independent basic mobility (CAS = 6; 57%) as compared to the placebo group (4.5 (range, 2-6); 36%). These findings on postoperative recovery are comparable with those from the previous study by Kang et al, as the mean time from surgery to when the patient started walking or standing and performing standing exercises was non-significantly better in the intervention group as compared to the control group (2.9 days versus 3.9 days, P = 0.067) [23]. According to the previous study by Kristensen et al., the median time from surgery to the return of independent basic mobility (CAS = 6) was seven days (interquartile range 4 to 11 days) [26]. This might imply that PMDI may accelerate early ambulation due to the significant improvement of VAS at the 48th hour postoperatively, which motivates those elderly hip fracture patients to get out of bed earlier than the placebo group. The results from the present study also showed a significant improvement after surgery (within the same group) and a non-significant difference in postoperative functional outcomes between both groups, as defined by the TUG test and HHS at two, six, and 12 weeks postoperatively (Table 7). This implied that the PMDI did not provide any significant benefits for short-term postoperative functional outcomes in the elderly femoral neck fracture patients who underwent bipolar hip arthroplasty.

Our study also had some limitations. First, we had a small sample size and a short-term follow-up period due to the outbreak of novel coronavirus (COVID-19), which limited the number of eligible patients in this study. Therefore, this requires a larger sample size to find any possible significant results or complications related to PMDI. However, the possible bias from the confounding factors was controlled by our research methodology as a triple-blinded RCT. Second, this study used bupivacaine in our PMDI regimen instead of ropivacaine as in previous studies. Ropivacaine is a long-acting local anesthetic that is structurally related to bupivacaine. It is a pure S(-) enantiomer, unlike bupivacaine, which is a racemate developed for the purpose of reducing potential toxicity and improving relative sensory and motor block profiles [27]. Therefore, the effectiveness of PMDI in this study might be affected by the efficacy of bupivacaine. Third, this study did not compare the outcome between PMDI with and without NSAIDs. Therefore, the actual benefits of NSAIDs in the PMDI regimen were not explored, and further research is required for the effectiveness of the PMDI without NSAIDs regimen in the elderly femoral neck fracture patients who undergo bipolar hip arthroplasty.

## Conclusions

PMDI with NSAIDs, comprising only 100-mg bupivacaine, 10-mg morphine sulfate, 300-mcg epinephrine, 750-mg cefuroxime, and physiologic saline, is an alternative for multimodal pain management in the elderly with a femoral neck fracture undergoing bipolar hip replacement, with a significant postoperative analgesic effect and possibly additional benefits on narcotic consumption on the second postoperative day.
